# Diversity of Lepidoptera (Insecta) recorded in a forest nursery of Nordeste County on São Miguel Island (Azores)

**DOI:** 10.3897/BDJ.10.e89971

**Published:** 2022-10-25

**Authors:** Virgílio Vieira, Luísa Oliveira, António Onofre Soares, Paulo A.V. Borges, Isabel Borges, João Tavares

**Affiliations:** 1 cE3c- Centre for Ecology, Evolution and Environmental Changes, Azorean Biodiversity Group, CHANGE – Global Change and Sustainability Institute, Faculty of Sciences and Technology, University of the Azores, Rua da Mãe de Deus, 9500-321, Ponta Delgada, Azores, Portugal cE3c- Centre for Ecology, Evolution and Environmental Changes, Azorean Biodiversity Group, CHANGE – Global Change and Sustainability Institute, Faculty of Sciences and Technology, University of the Azores, Rua da Mãe de Deus, 9500-321 Ponta Delgada, Azores Portugal; 2 CBA - Biotechnology Centre of Azores, Faculty of Agricultural Sciences and Environment, University of the Azores, Faculty of Sciences and Technology, University of the Azores, Rua da Mãe de Deus, 9500-321, Ponta Delgada, Azores, Portugal CBA - Biotechnology Centre of Azores, Faculty of Agricultural Sciences and Environment, University of the Azores, Faculty of Sciences and Technology, University of the Azores, Rua da Mãe de Deus, 9500-321 Ponta Delgada, Azores Portugal; 3 cE3c- Centre for Ecology, Evolution and Environmental Changes, Azorean Biodiversity Group, CHANGE – Global Change and Sustainability Institute, Faculty of Agricultural Sciences and Environment, University of the Azores, Rua Capitão João d´Ávila, Pico da Urze, 9700-042, Angra do Heroísmo, Azores, Portugal cE3c- Centre for Ecology, Evolution and Environmental Changes, Azorean Biodiversity Group, CHANGE – Global Change and Sustainability Institute, Faculty of Agricultural Sciences and Environment, University of the Azores, Rua Capitão João d´Ávila, Pico da Urze, 9700-042 Angra do Heroísmo, Azores Portugal

**Keywords:** Lepidoptera, Argyresthiidae, Crambidae, Erebidae, Geometridae, Noctuidae, Sphingidae, Tineidae, Tortricidae, Azores Islands

## Abstract

**Background:**

The diversity of moth species (Insecta, Lepidoptera) recorded in the forest nursery of Nordeste County on São Miguel Island (Azores) is given. Adults were sampled between March and December 2019 using three methods: (i) light trap to catch Noctuidae species, (ii) open-sided delta trap baited with a synthetic female sex pheromone lure to attract *Epiphyaspostvittana* (Walker) males and (iii) entomological net to collect microlepidopteran moths. This contribution focuses mainly on the diversity of moths present in one forest nursery of Nordeste County of São Miguel Island (Azores), especially on the species associated with endemic and native plant species. It also contributes to better plan strategies for integrated protection and conservation measures, since nurseries host a great diversity of plants from the Laurel Forest, which may attract many lepidopteran species.

**New information:**

A total of 10160 adults belonging to 33 lepidopteran species were recorded and listed by families, including: Argyresthiidae, one species (3%), Crambidae, four species (12%), Erebidae, one species (3%), Geometridae, five species (15%), Noctuidae, 18 species (55%), Sphingidae, one species (3%), Tineidae, one species (3%) and Tortricidae, two species (6%). The families Noctuidae, Geometridae and Crambidae were the most diverse. Those with the highest abundance of adults were the Noctuidae family, followed by the Geometridae, Crambidae, Tortricidae and Tineidae. The number of caught adults was consistently higher during spring and summer, decreasing sharply in late autumn. For 13 species caught in the light trap, the adult sex ratio was favourable to females. An analysis of the colonisation status, feeding and primary hosts of these endemic, native or exotic moth species contributes to our understanding of the factors that may lead to their establishment in Laurel Forest environments and to what extent there is a need to monitor and control them mainly with biological control agents.

## Introduction

The Laurel Forests of these islands have been significantly impacted by human activities, mainly due to dramatic land-use changes (only about 5% of the original forests remain; Triantis et al. 2010), habitat degradation and the introduction of exotic and invasive species (Cardoso et al. 2010, Triantis et al. 2010, Borges et al. 2013, Terzopoulou et al. 2015).

Currently, the Official Forestry Services rear endemic and native Azorean plant species to seek the restoration of the Laurel Forest and areas with high erosion risk or sensitive from the hydrological point of view, to promote awareness-raising activities and support forestation by private landowners (Rosagro et al. 2019). To this end, in São Miguel Island, thousands of the endemic and native plants are annually reared in two forest nurseries located in Povoação (Furnas) and Nordeste Counties. The plants reared in these forest nurseries are highly susceptible to attack by insect pests, such as aphid and lepidopteran species (Rosagro et al. 2019).

According to the most recent list of terrestrial organisms of the Azores (updated from Borges et al. 2010, Vieira et al. 2021), arthropods are the most diverse taxon, both in absolute richness (about 2364 taxa) and in number of endemic species and subspecies (about 274) (updated from Borges et al. 2010). Nevertheless, most of the arthropods (58%; Borges et al. 2005) are considered introduced. Amongst the most diverse insects, we found the butterflies and moths (Lepidoptera) with 158 species and subspecies, including 40 endemics (updated from Borges et al. 2010, Vieira et al. 2021). About one third of Lepidoptera were considered as being in the Azores as a consequence of human introductions (Vieira and Karsholt 2010), in which snout moths (Pyralidae), fungus moths (Tineidae) and leaf-rollers (Tortricidae) are the families with the highest number of introduced taxa. Furthermore, at the same time that the number of Azorean endemic species has continued to increase with new species descriptions (e.g. Wagner 2014, Wagner 2015, Pérez Santa-Rita et al. 2020), some exotic species have also been recorded for the islands (Wagner and Hausmann 2014, Pérez Santa-Rita et al. 2018, Vieira 2020a, Vieira 2020b).

Most studies about Lepidoptera from the Azores focus on species description, ecology and distribution, providing crucial information for the conservation of this taxon (e.g. Wagner 2014, Wagner 2015, Borges et al. 2018). However, there is still poor knowledge about the potential risk of most Microlepidoptera as pests to their host plants, as well as the colonisation status of many moth species.

## General description

### Purpose

This contribution focuses mainly on the diversity of moths present in one forest nursery of Nordeste County of São Miguel Island (Azores), especially species associated with endemic and native plant species. It also contributes to better plan strategies of integrated protection and conservation measures, since nurseries host a great diversity of plants from the Laurel Forest, which may attract many lepidopteran species.

## Project description

### Title

Diversity of Lepidoptera recorded in a forest nursery of Nordeste County on São Miguel Island (Azores)

### Personnel

Virgílio Vieira, Luísa Oliveira, António O. Soares, Paulo A. V. Borges, Isabel Borges, João Tavares

### Study area description

The current study was performed in São Miguel Island (ca. 750 km^2^), the largest in the Archipelago of the Azores, located in the North Atlantic, roughly between the coordinates 37°54'38'' to 37°42'13''N latitude and 25°08'03'' to 25°51'17''W longitude. The climate is temperate oceanic, with regular and abundant rainfall, high levels of relative humidity and persistent winds, mainly during the winter and autumn seasons. The study was conducted in the Nordeste Nursery Recreational Forest Reserve located in Nordeste County (37°49'43.9"N, 25°08'59.5"W) at about 180 metres of altitude.

### Design description

Data from pheromone trap, light trap and entomological net sampling were analysed to assess differences in species richness, abundance and phenology of lepidopteran species and families. Adult sex ratio expressed as percentage of females was calculated for 14 Noctuidae species captured in the light trap following the formula: % females = (number of females/total of adults) x 100. All statistical analysis were performed using SPSS Statistics v. 27 software.

### Funding

Regional funds, through Official Forestry Services from Regional Government of the Azores, within the research project MoCIL “Monitorização e Controlo Integrado de Lepidópteros em Viveiros Florestais (Nordeste e Furnas) na ilha de São Miguel - Açores”, FCT – Fundação para a Ciência e a Tecnologia, I.P., under the project UIDP/05292/2020 and UIDB/05292/2020 and AZORESBIOPORTAL–PORBIOTA (ACORES-01-0145-FEDER-000072) (2019-2022).

## Sampling methods

### Study extent

The study covers the nocturnal lepidopterans that attack endemic and native Azorean forest plants reared in the nursery.

### Sampling description

Samples of adult moths were collected between March and December 2019 (i.e. 38 weeks), as generally few lepidopteran species are active during winter. Adults were monitored from dusk (18:00 h) to dawn (06:00 h) using an omnidirectional light trap, equipped with a TLD 18W lightbulb, according to the methodology used by Tavares (1989). Additionally, the *Epiphyaspostvittana* population was monitored from May to December 2019 (i.e. 31 weeks), using open-sided delta traps baited with a synthetic female sex pheromone lure (containing: E11-14Ac, E9E11-14Ac) to attract males (see Oliveira et al. 2022). The light trap and open-sided delta trap, spaced 300 m apart, were installed one metre over the ground level at the edge of the nursery for Azorean endemic forest plants, which included *Ericaazorica*, *Vacciniumcylindraceum* (both Ericaceae), *Ilexazorica* (Aquifoliaceae), *Juniperusbrevifolia* (Cupressaceae), *Laurusazorica* (Lauraceae), *Viburnumtreleasei* (Adoxaceae), *Prunusazorica* (Rosaceae), *Picconiaazorica* (Oleaceae), *Frangulaazorica* (Rhamnaceae) and one native species, *Morellafaya* (Myricaceae), which are listed in the IUCN Red List (see Rosagro et al. 2019, Vieira et al. 2020).

Throughout the study, adults were collected weekly in both trap types and lures changed once a month. In addition, the plants reared in the forest nursery were sampled once a week to collect microlepidopteran moths using a standard entomological net (35 cm diameter, 140 cm handle), which were immediately identified and released on site if the specimens did not need to be observed more carefully in the laboratory. Sweeping occurred during day hours.

### Quality control

All individuals caught in the traps were first sorted by Virgílio Vieira, João Tavares or Luisa Oliveira. Current taxonomic affiliation follows Vives-Moreno (Vives-Moreno 2014).

## Geographic coverage

### Description

The study was conducted in the Nordeste Nursery Recreational Forest Reserve located in Nordeste County (37°49'43.9"N, 25°08'59.5"W) at about 180 metres altitude.

### Coordinates

37°46'19.2'' and 37°49'55.2''N Latitude; 25°8'16.8'' and 25°15'3.6''W Longitude.

## Taxonomic coverage

### Description

The sampling programme targeted lepidopteran species (Insecta: Lepidoptera)

### Taxa included

**Table:** 

Rank	Scientific Name	Common Name
family	Lepidoptera	Moths

## Temporal coverage

### Notes

21 March 2019 - 12 December 2019

## Usage licence

### Usage licence

Creative Commons Public Domain Waiver (CC-Zero)

## Data resources

### Data package title

Diversity of Lepidoptera recorded in a forest nursery of Nordeste County on São Miguel Island (Azores)

### Resource link

http://ipt.gbif.pt/ipt/resource?r=lepidoptera_nordeste_smiguel

### Alternative identifiers

http://ipt.gbif.pt/ipt/resource?r=lepidoptera_nordeste_smiguel&v=1.8

### Number of data sets

2

### Data set 1.

#### Data set name

Table of Sampling Events

#### Data format

Darwin Core Archive

#### Character set

UTF-8

#### Download URL

http://ipt.gbif.pt/ipt/resource?r=lepidoptera_nordeste_smiguel&v=1.8

#### Data format version

version 1.8

#### Description

The following data include all the records for which a taxonomic identification of the species was possible. The dataset submitted to GBIF (Global Biodiversity Information Facility) is structured as a sample event dataset, with two tables: in the current event table, the data in this sampling event resource have been published as a Darwin Core Archive (DwCA), which is a standardised format for sharing biodiversity data as a set of one or more data tables. The core data file contains three records (eventID). This IPT (integrated publishing toolkit) archives the data and thus serves as the data repository. The data and resource metadata are available for download from Vieira et al. (2022).

**Data set 1.** 

Column label	Column description
eventID	Identifier of the events, unique for the dataset.
stateProvince	Name of the region of the sampling site.
islandGroup	Name of archipelago.
island	Name of the island.
country	Country of the sampling site.
countryCode	ISO code of the country of the sampling site.
municipality	Municipality of the sampling site.
locality	Name of the locality.
verbatimLocality	The original textual description of the place.
minimumElevationInMetres	The upper limit of the range of elevation (altitude, usually above sea level), in metres.
samplingProtocol	The sampling protocol used to capture the species.
decimalLatitude	Approximate centre point decimal latitude of the field site in GPS coordinates.
decimalLongitude	Approximate centre point decimal longitude of the field site in GPS coordinates.
geodeticDatum	The ellipsoid, geodetic datum or spatial reference system (SRS) upon which the geographic coordinates given in decimalLatitude and decimalLongitude are based.
coordinateUncertaintyInMetres	Uncertainty of the coordinates of the centre of the sampling plot.
coordinatePrecision	Precision of the coordinates.
georeferenceSources	A list (concatenated and separated) of maps, gazetteers or other resources used to georeference the Location, described specifically enough to allow anyone in the future to use the same resources.
habitat	The habitat of the sample.
samplingEffort	The numeric amount of time spent in each sampling.
eventDate	Date or date range the record was collected.
year	Year of the event.
sampleSizeValue	A numeric value for a measurement of the size (time duration, length, area or volume) of a sample in a sampling event.
sampleSizeUnit	The unit of measurement of the size (time duration, length, area or volume) of a sample in a sampling event.

### Data set 2.

#### Data set name

Table of Species Occurrence

#### Data format

Darwin Core Archive

#### Character set

UTF-8

#### Download URL

http://ipt.gbif.pt/ipt/resource?r=lepidoptera_nordeste_smiguel&v=1.8

#### Data format version

version 1.8

#### Description

The following data include all the records for which a taxonomic identification of the species was possible. The dataset submitted to GBIF (Global Biodiversity Information Facility) is structured as a sample event dataset, with two tables: in the current event table, the data in this sampling event resource have been published as a Darwin Core Archive (DwCA), which is a standardised format for sharing biodiversity data as a set of one or more data tables. The core data file contains 33 records (occurrenceID). This IPT (integrated publishing toolkit) archives the data and thus serves as the data repository. The data and resource metadata are available for download from Vieira et al. (2022).

**Data set 2.** 

Column label	Column description
eventID	Identifier of the events, unique for the dataset.
type	Type of the record, as defined by the Public Core standard.
licence	Reference to the licence under which the record is published.
institutionID	The identity of the institution publishing the data.
collectionID	The identity of the collection publishing the data.
institutionCode	The code of the institution publishing the data.
collectionCode	The code of the collection where the specimens are conserved.
datasetName	Name of the dataset.
basisOfRecord	The nature of the data record.
occurrenceID	Identifier of the record, coded as a global unique identifier.
recordedBy	A list (concatenated and separated) of names of people, groups or organisations who performed the sampling in the field.
identifiedBy	A list (concatenated and separated) of names of people, groups or organisations who assigned the Taxon to the subject.
organismQuantity	Number of individuals.
organismQuantityType	The type of quantification system used for the quantity of organisms.
lifeStage	The life stage of the organisms captured.
establishmentMeans	The process of establishment of the species in the location, using a controlled vocabulary: 'native', 'introduced', 'endemic', "uncertain".
dateIdentified	The date on which the subject was determined as representing the Taxon.
scientificName	Complete scientific name including author and year.
kingdom	Kingdom name.
phylum	Phylum name.
class	Class name.
order	Order name.
family	Family name.
genus	Genus name.
specificEpithet	Specific epithet.
infraspecificEpithet	Subspecific epithet.
taxonRank	Lowest taxonomic rank of the record.
scientificNameAuthorship	Name of the author of the lowest taxon rank included in the record.

## Additional information

### Results and Discussion

The present paper deals with the lepidopteran fauna that have been recorded during fieldwork at Nordeste County in São Miguel Island. Throughout the 38 weeks’ trials, a total of 10160 adults belonging to 33 lepidopteran species were recorded and listed alphabetically by families, including one species of Argyresthiidae (3%), four of Crambidae (12%), one of Erebidae (3%), five of Geometridae (15%), 18 of Noctuidae (55%), one of Sphingidae (3%), one of Tineidae (3%) and two of Tortricidae (6%) (Table 1). Amongst the most representative families in number of species are Noctuidae, Geometridae and Crambidae, respectively (Table 2). The Noctuidae family has the greatest abundance of individuals (9584; 94.33%) followed by Crambidae (286; 2.81%), Tineidae (129; 1.27%) and Tortricidae (196; 1.04%), while Sphingidae was the least abundant family (4; 0.04%) (Table 2).

**Figure 1.**
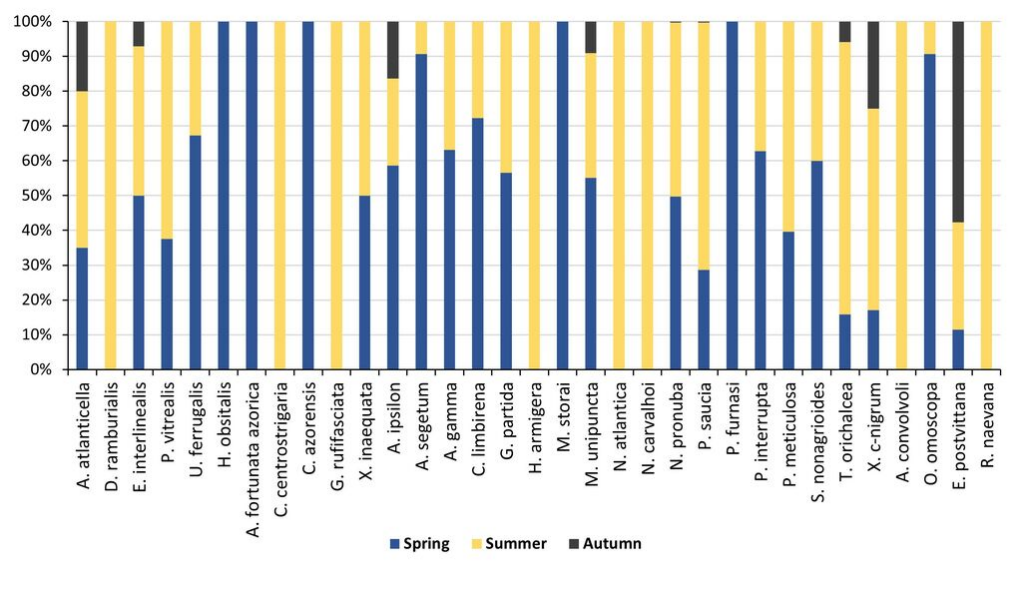
Seasonal relative abundance of adult moth species (%) collected in the nursery of Nordeste County, during 2019.

**Figure 2.**
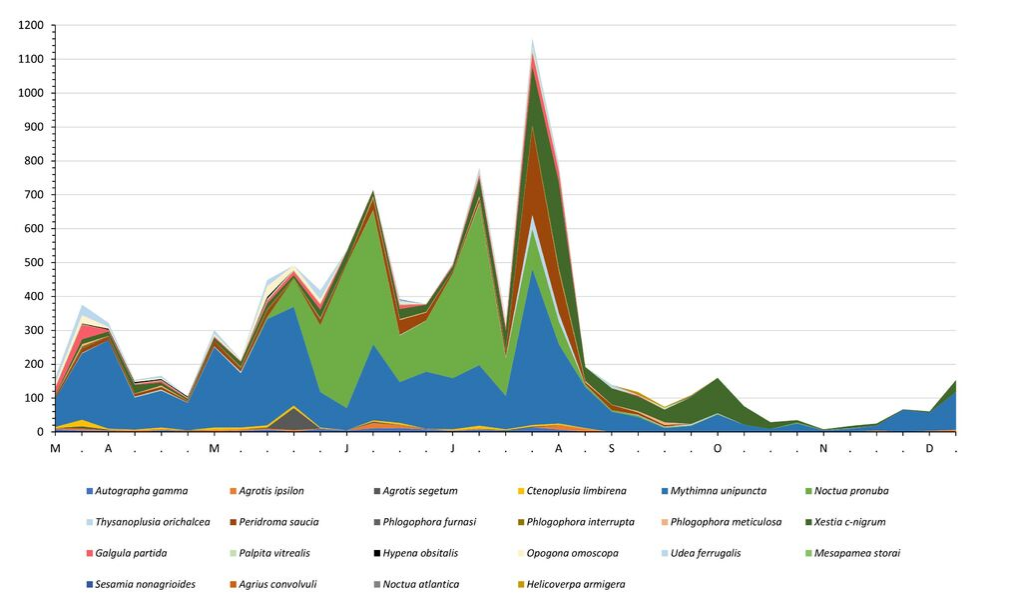
Weekly abundance of adult Lepidoptera species captured in the light trap in Nordeste County between March and December 2019. M - March, A - April, M - May, J - June, J - July, A - August, S - September, O - October, N - November, D - December.

The number of trapped adults was consistently higher during spring and summer, decreasing sharply in late autumn (Table 1, Figs 1, 2). Of the 33 species sampled, nine were recorded continuously during all three seasons (spring, summer and autumn), 11 for spring and summer and five species exclusive to spring and eight to summer (Table 1, Fig. 1).

In S. Miguel Island, 108 species of moth species are known. Fig. 2 shows that, from 33 moth’s species, 22 were caught in the light trap, mainly Noctuidae, which are amongst the most common constant in the Azorean Archipelago (e.g. Vieira and Karsholt 2010). The mean of individuals (± SE) recorded throughout the 35 weeks of the sampling period are as followed: *Autographagamma* (3.26 ± 0.66), *Agrotisipsilon* (2.97 ± 0.64), *Agrotissegetum* (2.77 ± 1.85), *Ctenoplusialimbirena* (2.69 ± 0.65), *Mythimnaunipuncta* (121.57 ± 17.69), *Noctuapronuba* (71.49 ± 22.43), *Noctuaatlantica* (0.03 ± 0.03), *Thysanoplusiaorichalcea* (2.89 ± 1.34), *Peridromasaucia* (18.97 ± 7.90), *Phlogophorafurnasi* (0.26 ± 0.12), *Phlogophorainterrupta* (1.23 ± 0.33), *Phlogophorameticulosa* (1.51 ± 0.37), *Xestiac-nigrum* (36.71 ± 8.65), *Galgulapartita* (6.97 ± 1.92), *Mesapameastorai* (0.03 ± 0.03), *Sesamianonagrioides* (0.14 ± 0.08), *Helicoverpaarmigera* (0.31 ± 0.19) (Noctuidae), *Agriusconvolvuli* (0.11 ± 0.09) (Sphingidae), *Palpitavitrealis* (2.12 ± 0.49), *Udeaferrugalis* (5.69 ± 1.46) (Crambidae), *Hypenaobsitalis* (0.54 ± 0.22) (Erebidae) and *Opogonaomoscopa* (3.63 ± 1.20) (Tineidae).

The most abundant noctuid species were *M.unipuncta* (41.88%), *N.pronuba* (24.63%), *X.c-nigrum* (12.65%), *P.saucia* (6.54%) and *G.partita* (2.40%), as had been observed in previous studies (Silva et al. 1994, Silva et al. 1995, Silva et al. 1995a, Silva et al. 1995b, Vieira et al. 2004), while the least abundant included, for example, the endemic species *M.storai*, *N.atlantica* and *N.carvalhoi*, accounting for only 0.01% of the total adults sampled (Table 1; Fig. 2).

Weekly abundance of the most frequent nocturnal lepidopterans were similar to those observed in previous studies conducted in the Azores (e.g. Tavares 1989, Carvalho et al. 1999, Vieira et al. 2004). In particular, for *M.unipuncta*, it was observed that, after three remarkable peaks of captures in early July, August and September, there was a steady decline to the lowest values in mid-October, in addition to the low number of adults captured (nil in some weeks) in early winter and spring. The number of adults captured varies significantly depending on the type of trap (light trap or sex pheromone trap), location and season (Vieira et al. 2004). The collected moths were from the adult stage, but larvae of the most abundant species do not feed on woody plants.

Concerning sex ratio, for 13 moth species caught in the light trap, it was biased towards females, namely: *A.gamma* (65%), *A.ipsilon* (67%), *A.segetum* (63%), *C.limbirena* (67%), *M.unipuncta* (60%), *N.pronuba* (62%), *T.orichalcea* (62%), *P.saucia* (65%), *P.interrupta* (70%), *P.meticulosa* (57%), *H.armigera* (55%), *S.nonagrioides* (60%) and *A.convolvuli* (75%). The biased ratio of females to males in these species may occur periodically and is probably related to their non-seasonal migratory movements (see also Silva et al. 1994, Silva et al. 1995a, Silva et al. 1995b, Vieira et al. 2004).

**Figure 3.**
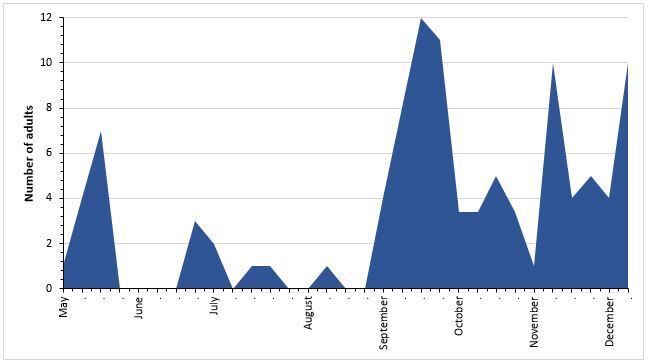
Males of *Epiphyaspostvittana* caught weekly in the sex pheromone trap installed in Nordeste Nursery between May and December 2019.

Regarding *E.postvittana*, 104 males were captured in the sex pheromone trap (Table 1; Fig. 3), corresponding to the mean (±SE) weekly number of 3.33 ± 0.65 individuals. According to Oliveira et al. (2022), this number was lower than that observed at Furnas (N = 300; 9.68 ± 1.98) over the same sampling period, although the annual pattern of adult distribution was relatively similar for both sampling sites. The continuous presence of the moth larvae year-round, complemented with the information resulting from the captures of adults in sex pheromone traps, allowed us to identify the light brown apple moth, *E.postvittana*, as a major lepidopteran pest that attacks endemic and native plants of the Azorean Forest (Oliveira et al. 2022).

Sampling of Microlepidoptera moths on young forest plants reared in the Nordeste Nursery using an entomological net showed that the most common moths are endemic species that appear associated with their known host plants (Table 3) (see Silva et al. 1995, Nuss et al. 1997, Wagner 2014, Wagner 2015, Borges et al. 2018, Pérez Santa-Rita et al. 2018). *Argyresthiaatlanticella* is the second most common lepidopteran in the Azores (Peres Santa-Rita et al. 2020), but in the nurseries, they have population densities with low numbers of individuals (Table 1), which is, amongst other factors, related to their nocturnal (rather than diurnal) activity and some of these species are effectively rare or have a conservation status of vulnerable.

Table 3 shows that 33 lepidopterans recorded are classified with the colonisation status of endemic (10 species), native (16), introduced (4) and introduced/naturalised (3). Regarding their feeding mode, 23 are generalists or polyphagous and 10 are specialists and the larvae can feed on plants of the Laurel Forest (14 species), endemic / native (23 species) and non-native (23 species).

According to previous studies and our field observations, many of the moth species recorded in this study (see Tables 1, 3) have little economic impact in the Azores Archipelago, but others may constitute a potential risk as pests (e.g. species from families of Noctuidae, Tortricidae and Crambidae) of introduced/naturalised and/or endemic/natives plants. For example, in contrast to the low number of *Palpitavitrealis* adults caught in the light trap (Table 1, Fig. 2), its larvae were observed to strongly and exclusively attack *Picconiaazorica* plants grown at the Nordeste Nursery and could reach 16.3% of the plants sampled (cf. Oliveira et al. 2022). On the other hand, *Cyclophoraazorensis* larvae attacks their host plant *Ericaazorica*, while *Argyresthiaatlanticella* larvae feed on *Ericaazorica*, *Vacciniumcylindraceum* and *Morellafaya* male flowers and green fruits and occur both in Laurel Forest habitats and in different types of habitats disturbed with *Morella*, *Erica* or *Vaccinium*. Additionally, *A.fortunataazorica* preferentially feeds on *Morellafaya* (e.g. Silva et al. 1995, Nuss et al. 1997, Wagner 2014, Wagner 2015, Borges et al. 2018, Pérez Santa-Rita et al. 2020). Other studies (Ribeiro et al. 2005, Rego et al. 2019) show that these species are associated with different hosts (e.g. *A.fortunata* on *E.azorica*), but neither shows that their impact severely affects plant fitness/reproduction. In addition, herbivore abundance may match host plant abundance, not being necessarily a pest.

### Conclusions

Our results provide information on the diversity of moths (Insecta, Lepidoptera) present in Nordeste County, whose adults were sampled in the Nordeste Forest Nursery. A total of 10160 adults belonging to 33 lepidopteran species were recorded and listed by families, including: Argyresthiidae, Crambidae, Erebidae, Geometridae, Noctuidae, Sphingidae, Tineidae and Tortricidae.

In general, the temporal profile of the abundance of adults caught in a light trap and a sex pheromone trap reveals that the Noctuidae, followed by the families Geometridae and Crambidae, have the highest species diversity and that these have high population densities during spring and summer, decreasing sharply in late autumn. In addition, for 13 species caught in the light trap, the adult sex ratio was favourable to females.

From the literature and our field observations, we conclude that many of the moth’s species recorded have little economic importance in the Azores Archipelago, but others may constitute a potential risk as pests (e.g. species from families of Noctuidae, Tortricidae and Crambidae) of introduced/naturalised and/or endemic/natives plants. In fact, the high abundance of adult moths observed for some species whose larvae feed preferentially on native and/or exotic herbaceous plants does not generally represent very serious damage to endemic and native forest plants of the Azores.

Some biological control agents are present in the field, for example, parasitising the larvae of *E.postvittana* (i.e. Braconidae species of *Meteorusictericus* (Nees, 1811) and *Microgasteropheltes* Nixon, 1968) and of *M.unipuncta* (i.e. *Glyptapantelesmilitaris* Walsh, 1861), as well as preying on several Noctuidae larvae (e.g. *Calosomaolivieri* Dejean, 1831, Coleoptera, Carabidae).

An analysis of the colonisation status of the lepidopteran moth species and their feeding and primary host plants associated with the Laurel Forest, native or non-native Azorean plants, suggests that forest nurseries may help us to understand the establishment of lepidopteran moths (endemic, native or exotic species) in Laurel Forest environments.

Finally, more studies are needed to understand two fundamental objectives; first, to know the potential damage caused by moth species on endemic and native Azorean plants; second, to assess the conservation status of all these moth species and to advise on possible future research and conservation actions critical to the long-term survival of the most endangered species.

## Figures and Tables

**Table 1.** List of moth species sampled in Nordeste County and their overall and seasonal abundance during the study period. Legend: S = species richness per family, N = abundance, % = relative frequency.

**Moth Species (S)**	**N**	%	**Season (N)**					
			**Spring**	%	**Summer**	%	**Autumn**	%
**Argyresthiidae (1)**								
*Argyresthiaatlanticella* Rebel, 1941	20	0.20	7	0.14	9	0.20	4	0.50
**Crambidae (4)**								
*Diasemiopsisramburialis* (Duponchel, 1834)	1	0.01	0	0	1	0.02	0	0
*Eudoniainterlinealis* (Warren, 1905)	14	0.14	7	0.14	6	0.13	1	0.12
*Palpitavitrealis* (Rossi, 1794)	72	0.71	27	0.56	45	0.99	0	0
*Udeaferrugalis* (Hübner, 1796)	199	1.96	134	2.77	65	1.44	0	0
**Erebidae (1)**								
*Hypenaobsitalis* (Hübner, 1813)	19	0.19	19	0.39	0	0	0	0
**Geometridae (5)**								
*Ascotisfortunataazorica* Pinker, 1971	4	0.04	4	0.08	0	0	0	0
*Costaconvexacentrostrigaria* (Wollaston, 1858)	1	0.01	0	0	1	0.02	0	0
*Cyclophoraazorensis* (Prout, 1920)	4	0.04	4	0.08	0	0	0	0
*Gymnoscelisrufifasciata* (Haworth, 1809)	1	0.01	0	0	1	0.02	0	0
*Xanthorhoeinaequata* Warren, 1905	2	0.02	1	0	1	0.02	0	0
**Noctuidae (18)**								
*Agrotisipsilon* (Hufnagel, 1766)	104	1.02	61	1.26	26	0.57	17	2.12
*Agrotissegetum* (Denis & Schiffermüller, 1775)	97	0.95	88	1.82	9	0.20	0	0
*Autographagamma* (Linnaeus, 1758)	114	1.12	72	1.49	42	0.93	0	0
*Ctenoplusialimbirena* (Gueneé, 1852)	94	0.93	68	1.41	26	0.57	0	0
*Galgulapartita* Guenée, 1852	244	2.40	138	2.85	106	2.34	0	0
*Helicoverpaarmigera* (Hübner, 1808)	11	0.11	0	0	11	0.24	0	0
*Mesapameastorai* (Rebel, 1940)	1	0.01	1	0.02	0	0	0	0
*Mythimnaunipuncta* (Haworth, 1809)	4255	41.88	2346	48.5	1526	33.7	383	47.82
*Noctuaatlantica* (Warren, 1905)	1	0.01	0	0	1	0.02	0	0
*Noctuacarvalhoi* (Pinker, 1983)	1	0.01	0	0	1	0.02	0	0
*Noctuapronuba* (Linnaeus, 1758)	2502	24.63	1245	25.74	1251	27.7	6	0.75
*Peridromasaucia* (Hübner, 1808)	664	6.54	190	3.93	472	10.4	2	0.25
*Phlogophorafurnasi* Pinker, 1971	9	0.09	9	0.19	0	0	0	0
*Phlogophorainterrupta* (Warren, 1905)	43	0.42	27	0.56	16	0.35	0	0
*Phlogophorameticulosa* (Linnaeus, 1758)	53	0.52	21	0.43	32	0.71	0	0
*Sesamianonagrioides* (Lefèbvre, 1827)	5	0.05	3	0.06	2	0.04	0	0
*Thysanoplusiaorichalcea* (Fabricius, 1775)	101	0.99	16	0.33	79	1.75	6	0.75
*Xestiac-nigrum* (Linnaeus, 1758)	1285	12.65	219	4.53	744	16.45	322	40.20
**Sphingidae (1)**								
*Agriusconvolvuli* (Linnaeus, 1758)	4	0.04	0	0	4	0.09	0	0
**Tineidae (1)**								
*Opogonaomoscopa* (Meyrick, 1893)	129	1.27	117	2.42	12	0.27	0	0
**Tortricidae (2)**								
*Epiphyaspostvittana* (Walker, 1863)	104	1.02	12	0.25	32	0.71	60	7.49
*Rhopobotanaevana* (Hübner, 1817)	2	0.02	0	0	2	0.04	0	0
**Total**	**10160**	**100**	**4836**	**100**	**4523**	**100**	**801**	**100**

**Table 2.** Species richness, absolute and relative abundance of Lepidoptera families. Legend: S = species richness, N = abundance, % = relative frequency.

**Families**	**Taxa (S)**	%	**N**	%
Argyresthiidae	1	3	20	0.20
Crambidae	4	12	286	2.81
Erebidae	1	3	19	0.19
Geometridae	5	15	12	0.12
Noctuidae	18	55	9584	94.33
Sphingidae	1	3	4	0.04
Tineidae	1	3	129	1.27
Tortricidae	2	6	106	1.04
**Total**	**33**	**100**	**10160**	**100**

**Table 3.** Colonisation status, diet and group of host plants potentially attacked by the moth species. END = Endemic, NAT = Native, INT = Introduced, I(nat) = Introduced (naturalised), P = Polyphagous, S = Specialist.

**Species**	**Colonisation Status**	**Diet**	**Group of host plants**		
			**Laurel Forest (LF)**	**Endemic**/ **Native (N)**	**Not Native (NN)**
*Argyresthiaatlanticella* Rebel, 1941	END	P	LF	N	
*Diasemiopsisramburialis* (Duponchel, 1834)	NAT	P			NN
*Eudoniainterlinealis* (Warren, 1905)	END	S	LF	N	
*Palpitavitrealis* (Rossi, 1794)	NAT	P	LF	N	NN
*Udeaferrugalis* (Hübner, 1796)	NAT	P		N	NN
*Hypenaobsitalis* (Hübner, 1813)	NAT	S			NN
*Ascotisfortunataazorica* Pinker, 1971	END	P	LF	N	
*Costaconvexacentrostrigaria* (Wollaston, 1858)	NAT	P			NN
*Cyclophoraazorensis* (Prout, 1920)	END	S	LF	N	
*Gymnoscelisrufifasciata* (Haworth, 1809)	NAT	P	LF	N	NN
*Xanthorhoeinaequata* Warren, 1905	END	S	LF	N	
*Agrotisipsilon* (Hufnagel, 1766)	I(nat)	P		N	NN
*Agrotissegetum* (Denis & Schiffermüller, 1775)	I(nat)	P		N	NN
*Autographagamma* (Linnaeus, 1758)	NAT	P			NN
*Ctenoplusialimbirena* (Gueneé, 1852)	NAT	P			NN
*Galgulapartita* Guenée, 1852	NAT	S		N	NN
*Helicoverpaarmigera* (Hübner, 1808)	I(nat)	P			NN
*Mesapameastorai* (Rebel, 1940)	END	S	LF	N	
*Mythimnaunipuncta* (Haworth, 1809)	NAT	P		N	NN
*Noctuaatlantica* (Warren, 1905)	END	S	LF	N	
*Noctuacarvalhoi* (Pinker, 1983)	END	S	LF	N	
*Noctuapronuba* (Linnaeus, 1758)	NAT	P		N	NN
*Peridromasaucia* (Hübner, 1808)	NAT	P		N	NN
*Phlogophorafurnasi* Pinker, 1971	END	S	LF	N	
*Phlogophorainterrupta* (Warren, 1905)	END	S	LF	N	
*Phlogophorameticulosa* (Linnaeus, 1758)	NAT	P		N	NN
*Sesamianonagrioides* (Lefèbvre, 1827)	INT	P			NN
*Thysanoplusiaorichalcea* (Fabricius, 1775)	NAT	P			NN
*Xestiac-nigrum* (Linnaeus, 1758)	NAT	P		N	NN
*Agriusconvolvuli* (Linnaeus, 1758)	NAT	P			NN
*Opogonaomoscopa* (Meyrick, 1893)	INT	P			NN
*Epiphyaspostvittana* (Walker, 1863)	INT	P	LF	N	NN
*Rhopobotanaevana* (Hübner, 1817)	INT	P	LF	N	NN
